# Impfstoffe gegen Hepatitis E: Wo stehen wir?

**DOI:** 10.1007/s00103-022-03487-1

**Published:** 2022-01-31

**Authors:** Patrick Behrendt, Heiner Wedemeyer

**Affiliations:** Klinik für Gastroenterologie, Hepatologie und Endokrinologie, Medizinische Hochschule Hannover, Carl-Neuberg-Str. 1, 30625 Hannover, Deutschland

**Keywords:** Deutschland, Impfstrategie, Neutralisierende Antikörper, HEV-239, Risikogruppen, Germany, Vaccine strategy, Neutralizing antibodies, HEV-239, Risk groups

## Abstract

In Europa ist aktuell kein Impfstoff gegen das Hepatitis-E-Virus (HEV) zugelassen. Demgegenüber steht in China bereits seit 10 Jahren mit HEV-239 (Hecolin®, Xiamen Innovax Biotech Co., Xiamen, China) ein Vakzin gegen den HEV-Genotyp 4 zur Verfügung. Herausforderungen für die Entwicklung von Impfstoffen ergeben sich v. a. aus den Unterschieden zwischen den Genotypen bezüglich Verbreitung, Übertragungswege und Risikogruppen. Weitere Hindernisse sind die Umhüllung von HEV im Blut durch Wirtsmembranen, die Replikation in verschiedenen Organen außerhalb der Leber sowie schwächere Immunantworten in vulnerablen Gruppen. In diesem Artikel wird der aktuelle Stand der verfügbaren und in fortgeschrittener präklinischer Evaluation befindlichen Vakzine gegen HEV mit Fokus auf Strategien der Impfstoffentwicklung dargestellt. Herausforderungen und Limitationen werden beschrieben.

Aktuelle Impfkandidaten fokussieren auf proteinbasierte Immunisierungen mit dem Ziel der Induktion von schützenden, neutralisierenden Antikörperantworten. Das Ziel der HEV-239-Zulassungsstudie mit mehr als 100.000 Studienteilnehmern war die Verhinderung von akuten symptomatischen Infektionen. Es ist jedoch unklar, inwieweit asymptomatische Infektionen durch das Vakzin verhindert wurden und ob es in Risikopatienten für einen komplizierten Verlauf, wie Patienten mit Leberzirrhose, Immunsupprimierten und Schwangeren, effektiv genug wirkt. Effiziente In-vitro-Modelle ermöglichen zunehmend die Entwicklung von monoklonalen neutralisierenden Antikörpern zur passiven Immunisierung oder Therapie.

Zukünftige Vakzine sollten neben einem sehr guten Sicherheitsprofil eine eindeutige Protektion gegenüber allen Genotypen demonstrieren. Die Entwicklung einer effizienten passiven Immunisierungsstrategie, insbesondere für immunsupprimierte Personen, ist wünschenswert.

## Abstract (en)

No vaccine against the hepatitis E virus (HEV) is currently licensed in Europe. In contrast, HEV-239 (Hecolin®, Xiamen Innovax Biotech Co., Xiamen, China), a vaccine against HEV genotype 4, has been available in China for 10 years. Challenges for the development of vaccines arise mainly from the differences between the genotypes with regard to distribution, transmission routes and risk groups. Other obstacles include the envelopment of HEV in blood by host membranes, replication in various organs outside the liver and weaker immune responses in vulnerable groups. This article reviews the current status of vaccines against HEV that are available and in advanced preclinical evaluation, with a focus on vaccine development strategies. Challenges and limitations are described.

Current vaccine candidates focus on protein-based immunisation with the aim of inducing protective, neutralising antibody responses. The goal of the HEV-239 pivotal trial with more than 100,000 study participants was to prevent acute symptomatic infections. However, it is unclear to what extent asymptomatic infections were prevented by the vaccine and whether it is effective enough in patients at risk for a complicated course, such as patients with liver cirrhosis, immunosuppressed individuals and pregnant women. Efficient in vitro models are increasingly enabling the development of monoclonal neutralising antibodies for passive immunisation or therapy.

Future vaccines should demonstrate clear protection against all genotypes in addition to a very good safety profile. The development of an efficient passive immunisation strategy, especially for immunosuppressed individuals, is desirable.

## Einleitung

Das Hepatitis-E-Virus (HEV) ist ein weltweit vorkommendes Pathogen welches – entgegen unserem Wissen von vor ca. 15 Jahren – nicht nur eine reiseassoziierte Erkrankung ist, sondern auch eine Vielzahl an autochthon erworbenen Infektionen in den Industrieländern verursacht. Es wird in 4 hauptsächlich krankheitsverursachende Genotypen (GT1-4) und verschiedene Subtypen differenziert, die Unterschiede u. a. in Epidemiologie und Klinik aufweisen. In Europa ist aktuell kein Impfstoff gegen HEV zugelassen, während in China seit 10 Jahren mit HEV-239 (Hecolin®, Xiamen Innovax Biotech Co., Xiamen, China) ein Vakzin zur Verfügung steht, das mit hoher Effektivität das Risiko für eine symptomatische akute Hepatitis E des dort vorkommenden GT4 reduziert.

Bei der Entwicklung von Impfstoffen bestehen zahlreiche Herausforderungen, die sich v. a. aus den Unterschieden zwischen den Genotypen z. B. in Hinblick auf Verbreitung, Übertragungswege und Risikogruppen ergeben. Noch nicht ausreichend geklärt ist, inwieweit bestimmte Hindernisse adressiert werden können, wie beispielsweise die Umhüllung von HEV im Blut durch Wirtsmembranen, die Replikation in verschiedenen Organen außerhalb der Leber sowie schwächere Immunantworten in vulnerablen Gruppen nach einer Impfung.

In diesem Artikel möchten wir den aktuellen Stand der verfügbaren und in fortgeschrittener präklinischer Evaluation befindlichen Vakzine gegen HEV darstellen. Ein spezieller Fokus liegt hierbei auf der Darstellung aktueller Strategien der Impfstoffentwicklung, den bereits in klinischen Studien evaluierten Vakzinen sowie Herausforderungen und Limitationen der HEV-Impfstoffentwicklung.

## Rationale für die Entwicklung eines Impfstoffes gegen Hepatitis E

Insbesondere die schweren Verlaufsformen der durch die HEV-GT 1 und 2 ausgelösten Infektionen im Rahmen einer Schwangerschaft, welche eine Mortalität von bis zu 30 % verursachen, legen die Notwendigkeit einer Impfung dieser Personengruppe nahe [1]. Obgleich in den Industrieländern die Infektion mit Hepatitis E typischerweise mild verläuft, besteht insbesondere bei Patienten mit fortgeschrittener Leberfibrose und -zirrhose das Risiko der Entwicklung eines Leberversagens [2].

Gemäß Empfehlungen der Ständigen Impfkommission (STIKO) sollten Personen mit bekannter Lebererkrankung eine Impfung gegen Hepatitis A und B zum Schutze einer hepatischen Dekompensation erhalten (vgl. Epidemiologisches Bulletin 34/20 [3]). Eine ähnliche Argumentation würde sich für die Empfehlung einer HEV-Schutzimpfung ergeben [4]. Der in den westlichen Industrieländern vorkommende GT3 kann zudem bei immunsupprimierten Personen (insbesondere Personen nach Organtransplantation) zu einer chronischen Infektion und dadurch zu einer raschen Entwicklung einer Fibrose/Zirrhose der Leber führen [5]. Die Prävalenz von HEV-Infektionen in der Gruppe der organtransplantierten Personen in Deutschland und Europa ist bislang nicht klar. Publizierte Daten schwanken in der Punktprävalenz zwischen 0,15–1 % [6, 7]. Ein Schutz vor dieser Infektion durch eine Immunisierung, welche von der STIKO vor Transplantation für Hepatitis B empfohlen wird, wäre ergänzend sinnvoll.

Es gibt wenige Kalkulationen bzgl. des Kosten-Nutzen-Verhältnisses einer HEV-Impfung: Analysen für eine Region mit Prävalenz von GT1 und GT2 legen nahe, dass aufgrund der hohen allgemeinen Seroprävalenz keine universelle Impfung, sondern lediglich ein Screening auf den Antikörper Anti-HEV-IgG (Immunglobulin G) und gegebenenfalls nachfolgende Vakzinierung von Schwangeren am kosteneffektivsten wären [8]. Ähnliches wurde für die sporadische Infektion durch GT4 in China bei älteren Personen, welche ein Risiko für eine schwere Verlaufsform der Infektion haben, festgestellt [9, 10].

Zuletzt stellen Gebiete in Nordafrika und Asien Endemiegebiete für HEV dar, sodass eine Impfung von Reisenden in diese Gebiete eine sinnvolle Empfehlung sein könnte.

In Tab. 1 werden Personengruppen mit möglicher Indikation einer HEV-Impfung sowie Pro- und Kontraargumente zu deren Impfung aufgeführt.

**Table 1:** 

Personengruppe	Pro	Kontra
*Ältere Personen*	Erhöhtes Risiko einer schweren Verlaufsform der Infektion bei Älteren	Klare Altersgrenze in Studien bislang nicht definiert
*Personen mit Vorerkrankung der Leber bzw. Leberzirrhose*	Erhöhtes Risiko einer schweren Verlaufsform der Infektion bei lebervorerkrankten Personen. Ähnliche Argumentation wie für die Hepatitis-A- und Hepatitis-B-Impfung in dieser Patientengruppe	Impfantwort ggf. reduziert in dieser Personengruppe. Kosten-Nutzen-Verhältnis dieser Maßnahme unklar
*Personen vor Organtransplantation*	Schutz vor akuter und chronischer HEV-Infektion nach Transplantation. Ähnliche Argumentation wie für die STIKO-Empfehlung zur Impfung gegen Hepatitis B vor Transplantation	Langfristiger Impfschutz unter immunsuppressiver Therapie fraglich
*Reisende in Endemiegebiete*	Schutz vor Infektion durch kontaminiertes Wasser in Endemiegebieten. Ähnliche Argumentation wie für die Empfehlungen gegen Hepatitis A für Reisende	Kosten-Nutzen-Verhältnis dieser Maßnahme unklar
*Risikogruppen mit Kontakt zu infizierten Tieren*	Schutz vor Infektion durch infizierte Tiere	Andere Maßnahmen, wie z. B. verbesserte Handhygiene, ggf. ausreichend
*Schwangere/Frauen im gebärfähigen Alter*	Lebensbedrohliche Infektionen für GT1 und 2 beschrieben	Kosten-Nutzen-Verhältnis dieser Maßnahme unklar

*HEV* Hepatitis E Virus, *STIKO* Ständige Impfkommission, *GT* Genotyp

## Herausforderungen in der Entwicklung eines Impfstoffes gegen Hepatitis E

Eine Herausforderung in der Entwicklung und Analyse der Effektivität eines Impfstoffes gegen HEV ergeben sich aus den Unterschieden der GT, welche differente klinische Verlaufsformen der Infektion verursachen (Tab. 2). Während von den 4 hauptsächlich humanpathogenen Genotypen GT1 und GT2 zum Teil größere Ausbrüche durch kontaminiertes Wasser verursacht werden, führen GT3 und GT4 zu sporadischen Infektionen v. a. durch Konsum kontaminierter Fleischprodukte [11]. Dieses erschwert die klinische Prüfung und die Translation der Ergebnisse von Studien von einem GT auf den anderen. Darüber hinaus ist unklar, ob durch die genetischen Unterschiede eine Immunantwort auf einen Impfstoff auf der Grundlage eines GT auch einen Schutz vor anderen GT bewirkt.

**Table 2:** 

Herausforderung durch genotypische Unterschiede	Bedeutung für die Evaluation der Effektivität der Impfung
*Genetische Diversität des Virus*	Darstellung einer Protektion des Impfstoffes gegenüber allen Genotypen ist notwendig
*Unterschiedliche Übertragungswege*	Nachweis einer Protektion sowohl bei sporadischen Infektionen (GT3 und 4, primär durch kontaminierte Fleischprodukte) sowie HEV-Ausbrüchen (GT1 und GT2, durch kontaminiertes Wasser)
*Unterschiedliche Risikogruppen*	Darstellung einer Protektion des Impfstoffes von Schwangeren (GT 1 und GT2), immunsupprimierte Personen mit dem Risiko einer chronischen Verlaufsform (GT3, GT4 und Ratten-HEV) sowie Personen mit anderweitigen Lebergrunderkrankungen
*Weltweite Verbreitung des Virus*	Hohe Anforderungen in Bezug auf Stabilität/Lagerung der Vakzine, um entlegene Bereiche der Welt erreichen zu können

*HEV* Hepatitis E Virus, *GT* Genotyp

Weitere Schwierigkeiten bestehen in der Heterogenität des Verlaufes in verschiedenen Patientengruppen. In der Gruppe der Immunsupprimierten sind chronische Verlaufsformen gefürchtet; bei ihnen ist die Immunantwort abgeschwächt, sodass ein potenzieller Schutz durch ein Vakzin entsprechend in klinischen Studien gezielt geprüft werden sollte. Zudem stellen akute durch GT3 und GT4 verursachte HEV-Infektionen eher ein Risiko für ältere Personen dar, welche jedoch oft geringere Immunantworten auf Vakzine zeigen. Untersuchungen an Schwangeren sind aufgrund ethischer Implikationen nur bedingt durchführbar.

Wie auch in der Diskussion der aktuellen SARS-CoV-2-Impfstoffe ist eine klare Definition des Endpunktes von Studien zur Evaluation eines HEV-Impfstoffes notwendig. Hierbei ist z. B. bislang unklar, ob im Rahmen von HEV-Ausbrüchen durch kontaminierte Gewässer eine Impfung, welche lediglich vor einer symptomatischen Infektion schützt, ausreicht, um eine weitere Ausbreitung der Infektion in der Bevölkerung zu verhindern. HEV-Impfstoffe sollten daher im besten Fall eine sterilisierende Immunität induzieren und dies als Endpunkt einer Impfstoffstudie determiniert werden.

Nicht zuletzt sollte ein HEV-Vakzin stabil und einfach zu lagern sein, damit auch entlegene Bereiche der Welt, in denen insbesondere Ausbrüche von HEV auftreten, realistisch darauf zurückgreifen können.

## Grundlage und Fokus der Impfstoffentwicklung

Aktuell legen die bislang erhobenen Daten von HEV-Vakzinen nahe, dass eine humorale Immunantwort die Protektion gegenüber HEV determiniert. Hierbei stellt das Kapsidprotein des Virus (ORF2, „open-reading frame“) die entscheidende Zielstruktur von neutralisierenden Antikörpern dar. Der Aminosäuren(AS-)abschnitt der sog. P‑Domäne des ORF2 („protruding“, AS 462–606) spielt eine wichtige Rolle in der Interaktion des Virus mit der Wirtszelle und ist der Bindungsort bislang bekannter neutralisierender Antikörper [12, 13]. Entsprechend verfolgen Impfansätze die Expression von kurzen ORF2-Peptiden oder aber nutzen aus, dass ektop generierte Kapside sogenannte „virus like particles“ (VLP), also in ihrer Oberflächenstruktur dem eigentlichen Virus ähnliche Partikel, ausbilden können, welche hierdurch entsprechend zu einer dem Virus ähnlichen Konformation des Antigens beitragen.

## Klinische Studien zu HEV-239

**Figure 1:**
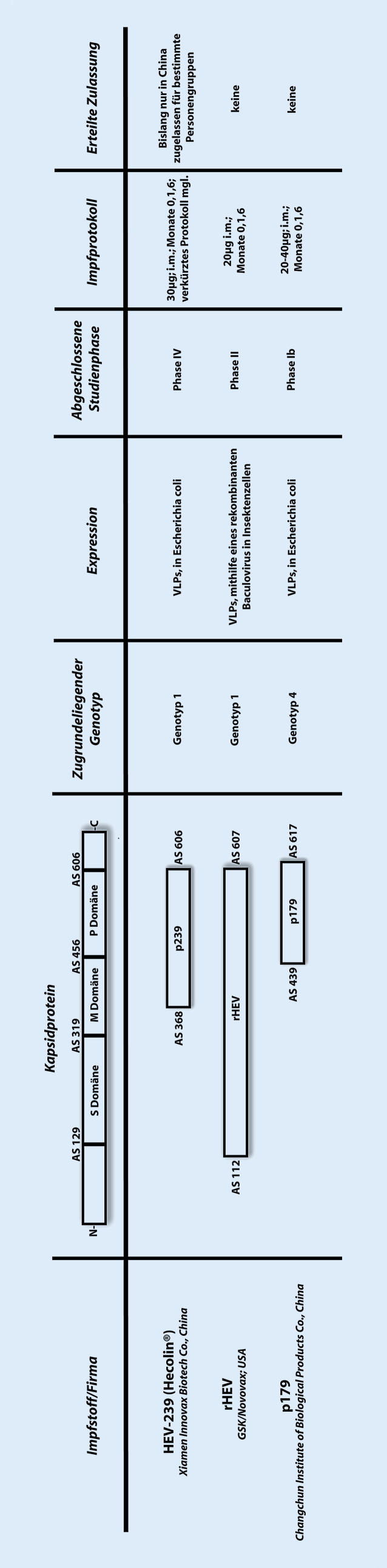

Das bislang einzige zugelassene Vakzin gegen HEV ist HEV-239 der Firma Xiamen Innovax Biotech Co., Ltd. in Xiamen, China (Abb. 1). Dieses ist seit Dezember 2011 durch die chinesische Behörde (China Food and Drug Administration) für Personen ab 16 Jahren mit dem Risiko einer HEV-Infektion (Personen mit engem Kontakt zu infizierten Tieren, Mitarbeiter der Armee, Studenten, in der Lebensmittelindustrie arbeitende Personen, Frauen im gebärfähigen Alter und Personen, welche in endemische Gebiete reisen) zugelassen.

HEV-239 ist ein 239 AS langes rekombinantes HEV-Peptid (auch HEV 239 bezeichnet), welches die AS 368–606 des Kapsidproteins des Virus (ORF2) beinhaltet [14]. Die AS eines GT1-HEV-Stammes wird hierbei in *E. coli* exprimiert, in denen es „inclusion bodies“ (Einschlusskörperchen) ausbildet. Das Peptid bildet nach Aufreinigung einen Homodimer mit einer Größe von 23 nm [15]. Basierend auf den Phase-II- und Phase-III-Studien erfolgt die intramuskuläre (i. m.) Applikation von HEV-239 insgesamt 3‑mal: Monat 0, 1 und 6. Hierbei besteht das Vakzin aus 30 µg Antigen in einer Mischung aus Natriumchlorid, Phosphat, Kalium Dihydrogenphosphat, Aluminiumhydroxid, Thiomersal und Wasser.

### Effektivität von HEV-239 im Menschen

Bislang liegen klinische Daten zur Effektivität von HEV-239 allein aus China vor, wo GT4 die hauptsächlich vorkommende GT-Variante des HEV darstellt. Die ersten Daten zur Effektivität von HEV-239 ergaben sich aus der Phase-II-Studie, welche hauptsächlich die Sicherheit und Immunogenität des Vakzins betrachtete. Als sekundärer Endpunkt wurden in dieser Studie auch HEV-Neuinfektionen untersucht [16]. Die Studie beinhaltete insgesamt 3 Arme: Gruppe A: Impfung mit 20 µg HEV-239 an Monaten 0, 1 und 6, *n* = 155; Gruppe B: Impfung mit 20 µg HEV-239 an Monaten 0 und 6, *n* = 151; Gruppe C: Hepatitis-B-Vakzin als Kontrolle an Monaten 0, 1 und 6, *n* = 151. Als Marker einer HEV-Infektion in einem Zeitraum von Monat 7–12 nach Impfung wurde eine spontane Serokonversion oder aber ein über 3facher Anstieg der Anti-HEV-IgG-Antikörper angesehen. In der Kontrollgruppe (Gruppe C) ergab sich in 9 von 104 Fällen der Hinweis auf eine HEV-Infektion, in Gruppe A in 1 von 102 und in Gruppe B in 1 von 78. Dieses entspricht einer Effektivität der Infektionsprävention von 85,2 % (95 % KI: 9,8–99,3) für Gruppe B und von 88,7 % (95 % KI: 31–99,5) für Gruppe A und damit insgesamt in Episoden pro Personen-Monaten einen signifikanten Unterschied zur Kontrollgruppe. Im Rahmen dieser Studien erfolgte eine Dosiseskalation an wenigen Probanden (3 × 10 µg, *n* = 45; 3 × 20 g, *n* = 49; 3 × 30 µg, *n* = 41; 3 × 40 µg, *n* = 20). Dabei führten alle Dosierungen zu einer 100 %igen Serokonversion der Probanden an Monat 12 nach Start der Immunisierung. Die Höhe der Anti-HEV-IgG-Titer zeigte einen dosisabhängigen Anstieg, dieser war jedoch nicht statistisch signifikant.

Auf Grundlage dieser Daten wurde die nachfolgende Phase-III-Studie mit Applikation von 30 µg HEV-239 durchgeführt [17]. Hierbei wurden für die doppelblinde, randomisierte Studie insgesamt 112.604 gesunde Erwachsene (16–65 Jahre) aus der Provinz Jiangsu in China zu gleichen Teilen aufgeteilt in die Vakzingruppe (56.302 Personen, Gabe HEV-239 i.m. 30 µg Monat 0, 1, 6) und die Kontrollgruppe (56.302, Gabe eines Vakzins gegen das Hepatitis-B-Virus [HBV] i.m. Monat 0, 1, 6). Beide Gruppen wurden für 9 Monate mit großem Aufwand engmaschig und aktiv auf die Entwicklung einer HEV-Infektion nachverfolgt und untersucht. Personen mit einer Hepatitis (definiert durch generelle Symptomatik für mehr als 3 Tage und Leberwertanstieg (Anstieg der Alanin-Aminotransferase [ALT] über das 2,5fache der Norm)) wurden auf unterschiedlichste hepatotrope Viren getestet. Die Diagnose einer akuten HEV-Infektion wurde bei Nachweis eines positiven HEV-IgM-Antikörpers, von HEV-RNA oder bei einem vierfachen Anstieg des Anti-HEV-IgG gestellt. In der „Per-Protokoll-Analyse“ ergaben sich in 15 von 48.663 Personen der Kontrollgruppe und in keiner der 48.693 mit HEV-239 behandelten Personen eine HEV-Infektion in der Zeit von einem Monat bis zu einem Jahr nach der dritten Impfung (Effektivität von 100 %; 95 % KI: 72,1–100; *p* < 0,0001). In der „Intention-to-treat-Analyse“ wurden die Studienteilnehmer zu Monat 19 analysiert, welche mindestens eine Impfung (HEV-239 oder Kontrollgruppe) erhalten hatten. In dieser ergab sich in 22 der 56.302 behandelten Personen aus der Kontrollgruppe und in einer (mit nur einer Impfung) der 56.302 HEV-239-behandelten Personen eine HEV-Infektion, was einer Effektivität von 95,5 % entspricht (95 % KI: 66,3–99,4 %; *p* < 0,0001).

Kürzlich wurden die Daten eines verkürzten Impfschemas von HEV-239 veröffentlicht. Hierbei wurde die Entwicklung einer HEV-Serokonversion nach Impfung mit HEV-239 nach Monat 0, 1 und 6 mit der verkürzten Immunisierung an Tag 0, 7 und 21 in einer randomisierten Studie an insgesamt 126 Probanden untersucht. Es ergab sich bei gleicher Nebenwirkungsrate auch eine gleiche Serokonversionsrate mit Entwicklung gleichwertiger Höhen der Antikörperantworten [18].

### Langzeitschutz nach HEV-239-Impfung

In der erweiterten Follow-up-Studie der ursprünglichen Phase-III-Studie wurden die Probanden für die Zeit nach der initialen Studie (19 Monate) bis 55 Monate nach erster Impfung weiter doppelblind nachverfolgt [19]. Dieses beinhaltete 56.022 der HEV-239-geimpften vs. 55.977 Personen aus der Kontrollgruppe. Es ergab sich bis zum Monat 55 eine nur minimale Lost-to-Follow-up-Rate auf 50.240 in der HEV-239- bzw. 55.185 in der Kontrollgruppe. Die Studienteilnehmer wurden angehalten bei Hinweisen auf eine Hepatitis (Gelbsucht, Abgeschlagenheit, epigastrische Schmerzen) einen Arzt aufzusuchen, woraufhin sie entsprechend der Hauptstudie klinisch und laborchemisch untersucht wurden. Insgesamt (zusammen mit den Infektionen vor dem 19. Monat) ergaben sich in der HEV-239-Gruppe in dem genannten Zeitraum 7 HEV-Infektionen. In der Kontrollgruppe waren es 53. Von den 7 der HEV-infizierten Personen in der HEV-239-Gruppe haben 3 alle, einer 2 und 3 weitere 1 Impfung erhalten. Die Effizienz der Impfung lag insgesamt bei 93,3 % (95 % KI: 78,6–97,9) in der „Per-Protokoll-Analyse“ und 86,8 % (95 % KI: 67,1–93,3) in der „Intention-to-treat-Analyse“. Die Infizierten in der HEV-239-Gruppe zeigten im Vergleich zu den Infizierten der Placebogruppe niedrigere ALT-Level (13,6fach vs. 23,3fach über der Norm), eine höhere Anti-HEV-IgG-Avidität (64 % vs. 6 %) sowie niedrigere Gesamt-Anti-HEV-IgM- und -IgG-Level. Von diesen Parametern zeigte sich allein die Avidität signifikant unterschiedlich. Eine weitere Analyse der Daten extrapolierte in Abhängigkeit von dem Berechnungsmodell, dass ca. 50 % der vakzinierten Personen nachweisbare Antikörpertiter für 8 bzw. > 30 Jahre behalten und somit für diesen Zeitraum wahrscheinlich geschützt sind vor einer HEV-Infektion [20].

### Verträglichkeit der HEV-239-Impfung

Im Rahmen der Phase-III-Studie ergaben sich signifikant mehr lokale Reaktionen im Bereich der Injektionsstelle in der HEV-239-Gruppe im Vergleich zur Kontrollgruppe (13,5 % vs. 7,1 %), welches am ehesten durch eine vermehrte Proteinmenge von HEV-239 im Vergleich zu dem HBV-Vakzin verursacht worden ist. Ansonsten war das Vakzin HEV-239 nebenwirkungsarm und sicher. Hauptbeschwerden waren Schmerz, Schwellung und Juckreiz im Bereich der Injektionsstelle. In einer Untergruppe von 1316 HEV-239- und 1329 HBV-immunisierten Personen fand eine aktive Nachverfolgung von Adverse Events (AE, dt.: unerwünschte Ereignisse, UE) bis 72 h nach Impfung statt. Die Rate der ≥ Grad-3-Reaktionen (Grad 0–5; Grad 0: keine Beschwerden, Grad 5: Versterben) war in beiden Gruppen gleich, sowohl für die lokalen (HEV-239: 0,2 %, Kontrollgruppe: 0,0 %; *p* = 0,248) als auch die systemischen Reaktionen (HEV-239: 0,5 %, Kontrollgruppe: 0,3 %; *p* = 0,356).

### Effektivität und Sicherheit der HEV-239-Impfung in bestimmten Patientenpopulationen

#### Schwangere Frauen.

Im Rahmen der Phase-III-Studie waren insgesamt 37 Frauen der HEV-239-Gruppe sowie 31 Frauen der Kontrollgruppe bei Einschluss oder während der Durchführung der Studie schwanger, obgleich dies eigentlich ein Ausschlusskriterium war. Hiervon waren 22 mit einer, 14 mit 2 und 1 mit 3 Dosen HEV-239 geimpft worden. Die Rate der AE war in beiden Gruppen gleich. Es gab keine Unterschiede hinsichtlich Abtreibungen oder spontaner Aborte. Keines der Neugeborenen hatte kongenitale Erkrankungen. Auch ergab sich kein Unterschied hinsichtlich Geburtsgewicht, Größe oder Gestationsalter der Frauen [21]. Aktuell werden in einer Phase-IV-Studie die Sicherheit, Verträglichkeit und Effektivität von HEV-239 bei Frauen im gebärfähigen Alter untersucht [22].

#### Effektivität und Sicherheit in Personen über 65 Jahre.

In einer „open-label“ (unverblindeten), kontrollierten Studie wurden die Sicherheit und die Immunogenität von HEV-239 in Personen über 65 Jahre mit Probanden im Alter von 18–65 Jahren verglichen. Hierbei ergab sich in Personen über 65 Jahren eine Serokonversion in 96,7 % zu Monat 7, welche vergleichbar war mit der Serokonversion in Personen im Alter zwischen 18 und 65 Jahren (97,1 %). In der Gruppe der zur Baseline (zu Beginn) seronegativen Teilnehmer war die mittlere Anti-HEV-IgG-Konzentration in den über 65-Jährigen 5,36 und in den 18- bis 65-Jährigen 10,84 WU (World Units)/mL zu Monat 7. Ein ähnliches Bild ergab sich bei den zur Baseline seropositiven Probanden: Hier führte die Impfung zu Monat 7 zu 19,65 WU/mL in der Altersgruppe über 65 und zu 24,52 WU/mL in der Gruppe der Personen zwischen 18 und 65 Jahren. Insgesamt waren die berichteten AE in den untersuchten Altersgruppen gleich [23]. Eine Studie mit identischem Umfang und gleicher Fragestellung wurde 2015 in der chinesischen Provinz Xiamen Haicang abgeschlossen, die Ergebnisse wurden bislang nicht veröffentlicht (ClinicalTrials.gov Identifier: NCT02189603).

### Aktuelle Studien mit HEV-239

Aktuell werden mehrere Studien durchgeführt, die mehrheitlich bereits abgeschlossen sind, deren Ergebnisse jedoch noch nicht präsentiert wurden.

NCT02584543 (ClinicalTrials.gov Identifier): In dieser „open-label“, randomisierten Phase-IV-Studie werden die Sicherheit und Immunogenität einer gleichzeitigen Impfung gegen HEV und HBV untersucht. Sie besteht aus insgesamt 3 Armen. Gruppe A erhält eine Kombinationsimpfung aus HEV-239 plus HBV-Impfung (*n* = 300), Gruppe B erhält HEV-239, Gruppe C erhält allein die HBV-Impfung (je 150).

NCT03365921: In dieser monozentrischen, doppelblinden, randomisierten Studie werden insgesamt 360 Personen in 3 Gruppen aufgeteilt und unterschiedliche Lots (Chargen) von HEV-239 verabreicht. Ziel ist hier der Ausschluss einer Lot-zu-Lot-Differenz bzgl. der Immunogenität.

NCT03827395: In dieser doppelblinden, randomisierten, placebokontrollierten Phase-I-Studie erhielten insgesamt 25 US-Amerikaner HEV-239 mit dem Ziel, die Sicherheit, Reaktivität und Immunogenität zu untersuchen.

NCT01735006: Dies ist eine Phase-III-Studie durchgeführt in China, in der ein neuer Impfstoff gegen das humane Papillomavirus (HPV) 16/18 untersucht wird. HEV-239 wird hierbei der Kontrollgruppe verabreicht, sodass weitere Daten bzgl. der Sicherheit bei Frauen im gebärfähigen Alter generiert werden.

## Klinische Studien weiterer Impfstoffe

2 weitere Impfstoffe sind bislang in klinischen Studien untersucht worden: rHEV und p179 (Abb. 1).

### rHEV

Das sogenannte rHEV-Vakzin ist eine proteinbasierte Impfung mit einem 56 kDa-Protein, welches den AS 112–607 des Kapsidproteins (ORF2) des GT1-HEV (Sar-55-Stamm) entspricht [24]. rHEV wurde initial durch das nationale Institut für Gesundheit (NIH) in den USA entwickelt und für klinische Studien durch die Firma DynCorp (heute Novovax) unter Sponsoring der US-Armee und GlaxoSmithKline produziert. rHEV wird mithilfe eines rekombinanten Baculovirus in Insektenzellen exprimiert. Die Immunogenität dieses Proteins wurde in präklinischer Phase in Makaken untersucht, in denen eine – wenn auch nicht sterilisierende – Immunität erreicht werden konnte [25].

In der Phase-I-Studie wurden insgesamt 88 US-Amerikaner zwischen 18 und 50 Jahren mit dem Protein in unterschiedlichen Dosierungen behandelt [26]. Darüber hinaus erfolgte eine Impfung von 44 Personen in Nepal mit 5 µg oder 20 µg Dosen (Monat 0, 1 und 6) von denen 43 bereits nach 2 Monaten und zu Monat 7 alle Teilnehmer der Studie eine Serokonversion zeigten [27]. In die nachfolgende Phase-II-Studie (randomisiert, placebokontrolliert) wurden in Nepal ca. 2000 gesunde, hauptsächlich männliche Erwachsene eingeschlossen [28]. Nach Gabe von insgesamt 3 Dosen je 20 µg des Vakzins (Monat 0, 1 und 6) ergab sich in einem medianen Follow-up von 804 Tagen eine Effektivität von 95,5 % für die Prävention einer HEV-Infektion. In den Personen, die mindestens eine Impfung erhalten haben, ergab sich eine Effektivität von 85,5 %. Das Vakzin war gut verträglich und es kam als Nebenwirkung vor allem zu lokalen Reaktionen im Bereich der Einstichstellen. Lediglich bei einem Probanden wurde dies als Schweregrad 3 (Grade 0–5) bewertet. Systemische Reaktionen gab es in beiden Gruppen (Vakzine und Placebo) je einmal (Fieber im Schweregrad 3). Trotz dieser positiven Ergebnisse erfolgte seither keine weitere klinische Prüfung des Impfstoffes.

### p179

Eine weitere Impfung gegen HEV ist in China in einer-Phase-I-Studie evaluiert worden. Das p179-Vakzine entspricht den AS 439–617 des GT4-HEV des Kapsidproteins (ORF2) und wird in *E. coli* exprimiert. Präklinische Daten zeigten, dass es nach Applikation von 5 µg zu der Entwicklung von protektiven Antikörpern gegenüber HEV in Makaken kommt. Die Gabe von 20 µg scheint vor einer Virämie experimentell infizierter Affen zu schützen. Diese Daten sind jedoch nur in der Veröffentlichung zur Phase-I-Studie des Vakzins erwähnt [29]. In dieser randomisierten, kontrollierten Phase-I-Studie wurden insgesamt 120 Personen im Alter zwischen 16 und 65 Jahren in der Provinz Jiangsu in China in die Kontrollgruppe (HEV-239) und die p179-Gruppe aufgeteilt. Die Applikation der Impfungen erfolgte hierbei erneut zu Monat 0, 1 und 6, zudem erfolgte eine Dosiseskalation von 20 µg, 30 µg und 40 µg des p179. Insgesamt wurden im Vergleich zur Kontrollgruppe weniger lokale Reaktionen beobachtet (*p* = 0,027). Die Gesamtheit der beobachteten AE mit systemischen Reaktionen war insgesamt bei beiden beobachteten Gruppen gleich und die Verträglichkeit gut. Daten zur Immunogenität im Menschen stehen bis dato aus. Eine Phase-II-Studie des p179-Vakzins wird aktuell durchgeführt [29].

## Impfungen in präklinischer Entwicklung

Eine Reihe weiterer proteinbasierter Vakzine befindet sich in präklinischen Phasen und wurde in nichtmenschlichen Primaten auf ihre Wirksamkeit untersucht. Es wurden jeweils unterschiedliche Anteile des Kapsidproteins als Vakzinkandidaten genutzt und in Affenmodellen untersucht. Eine Auflistung der bislang in präklinischer Entwicklung befindlichen Vakzine ist Tab. 3 zu entnehmen.

**Table 3:** 

Vakzin Name	Zugrunde liegende Impfstoffstrategie	Resultate der präklinischen Testung im Affenmodell
*trpE-C2-Vakzin*	GT1-Kapsid, AS 221–660, exprimiert in *E. coli*	Makaken konnten durch eine dreimalige Immunisierung mit 80 µg dieses Proteins sowohl vor einer Hepatitis durch einen homologen GT1 als auch vor einer heterologen Infektion mit GT2 geschützt werden. Hierbei ergab sich jedoch für GT2 der Nachweis einer Ausscheidung viraler RNA über den Stuhl der Tiere [48]
*62* *kDa-Vakzin*	GT1-Kapsid, AS 112–660, exprimiert in Insektenzellen	In Makaken zeigte sich in einem von 3 Tieren keine Protektion gegenüber einer Infektion mit einem GT2-Virusstamm aus Mexiko [49]
*53* *kDa-Protein Vakzin*	GT1-Kapsid, AS 112–578, exprimiert in Insektenzellen	Die Immunisierung von Makaken mit 2 Dosen des Impfstoffes konnte keine Protektion gegen eine Infektion mit dem homologen (GT1-)Virusstamm erzielen [50]
*T1-ORF2-Vakzin*	GT4-Kapsid, AS 126–621, exprimiert in CHO-Zellen („Chinese hamster ovary“)	Die Immunisierung von Makaken mit 2 40 µg-Dosen des Proteins führte zur Protektion gegenüber einer Infektion mit GT1 und GT4. Jedoch war dies abhängig von dem Titer des Inokulums, da eine höhere Viruslast zur Infektion der Tiere weiterhin zu einer manifesten Infektion führte [51]
*pcHEVORF2-Vakzin*	GT1, Plasmid-DNA-Vakzin (gesamte Kapsidsequenz)	Nach intramuskulärer Applikation von insgesamt 400 µg des Impfstoffes ergab sich nur in der Hälfte der immunisierten Makaken eine Protektion gegenüber dem mexikanischen HEV GT2. Unter Veränderung der Applikationsform zur Injektion per „gene gun“ konnte jedoch eine vollständige Protektion von Makaken vor einer Infektion mit einem GT2-Virusstamm erreicht werden [52]
*Lipo-NE-DP-Vakzin*	GT1, Plasmid-DNA-Vakzin, AS 458–607; zusammen mit dem entsprechenden Protein in E. coli exprimiert	Nach Immunisierung (2 Dosen, jeweils 20 µg DNA und 20 µg Protein) ergab sich eine Protektion der Tiere vor einer GT1-Virusinfektion [53]
*Bislang nicht benannt*	GT1 und GT3 Kapsid, AS 112–608, exprimiert in Insektenzellen	Es zeigte sich eine vollständige Protektion der Tiere gegenüber einer GT7-HEV-Infektion [54]
*rHEV-VLP-Vakzin (unterschiedlich zu Vakzin „rHEV“, welches sich in klinischer Testung befindet)*	GT1 Kapsid, AS 112–608, exprimiert in Insektenzellen	Die orale Applikation von 5 × 10 mg Dosen in Makaken schützte die Tiere vor einer GT1-Infektion, obgleich einige der Tiere weiter Virusausscheidung im Stuhl zeigten [55]

*GT* Genotyp, *AS* Aminosäuren, *DNA* Desoxyribonukleinsäure, *HEV* Hepatitis E Virus

Viele weitere Vakzinkandidaten befinden sich aktuell in Evaluation in Kleintiermodellen oder Charakterisierung *in vitro*. Weiterhin verfolgen diese Ansätze hauptsächlich die Generierung von Varianten des Kapsidproteins durch verschiedene Expressionssysteme, um eine humorale Antwort gegen HEV zu induzieren [30].

## Bewertung und zukünftige Herausforderungen der HEV-Vakzine

In ihrem Positionspapier vom Mai 2015 bewertet die Weltgesundheitsorganisation (WHO) HEV-239 als einen hoffnungsvollen weltweiten HEV-Vakzinkandidaten. Sie sieht die Notwendigkeit der Impfung von Personen mit erhöhtem Risiko für eine schwere Infektionsform, wie zum Beispiel Schwangere und Reisende. Jedoch werden die Daten insgesamt für die Empfehlung zur Routineimpfung als zu schwach angesehen. Insbesondere bemängelt die WHO fehlende Datensätze für Personen unter 16 Jahren, Schwangere, Personen mit chronischen Lebererkrankungen, Reisende und Personen auf der Warteliste für Transplantationen sowie transplantierte Personen unter Immunsuppression (Infobox 1*;* [31]).

Unklarheit besteht bezüglich des Schutzes durch HEV-239 vor anderen GT als GT4. Bislang wird angenommen, dass sämtliche GT einen gleichen Serotyp ausbilden und entsprechend eine antikörpervermittelte Protektion einen Schutz vor allen GT bringt, die eine relativ hohe genetische Ähnlichkeit des Kapsidproteins besitzen (85 %; [32]). Dass nur ein Serotyp besteht, stützt sich auf Daten von Konvaleszenten, deren Seren jeweilig die Kapsidproteine anderer GT *in vitro* bindet [33]. Und tatsächlich scheint dies zumindest für GT1 und GT4 der Fall zu sein, da hier das chinesische Vakzin (GT1-basiert) vor einer GT4-Infektion schützt. Wie jedoch weiter oben erwähnt, ergeben sich insbesondere in der Analyse von Vakzinkandidaten in Affenmodellen sehr unterschiedliche Ergebnisse bezüglich der Protektion vor heterologer Infektion. Auch zeigen Immunisierungsstudien mit den beiden Vakzinen HEV-239 und p179 ein deutlich heterogenes Bild der Immunantwort in Affenmodellen. So ergab sich für HEV-239 eine sehr starke Reaktivität der induzierten Antikörper gegen rekombinantes ORF2-Protein von GT1 und GT2 wobei sich für p179 vor allen Dingen eine Reaktivität gegen GT3 und GT4 darstellte [34]. Schwieriger wird es mit weiteren HEV-Virusstämmen, die zunehmend als humanpathogene Erreger identifiziert werden und phylogenetisch deutlich weiter von den anderen HEV-Stämmen entfernt sind. So weist das Kapsidprotein des Ratten-HEV, welches insbesondere in China als humanpathogener HEV-Stamm identifiziert wurde, nur eine ca. 48 %ige Homologie auf Aminosäureebene mit den anderen humanpathogenen HEV-Stämmen auf. Daher erscheint eine Protektion gegenüber dem Ratten-HEV durch einen Vakzinkandidaten, der auf einem anderen GT basiert, fraglich [35].

Ein weiterer Kritikpunkt ergibt sich aus der Schwierigkeit, dass die HEV-Infektion in China mit GT4 typischerweise milde und spontan selbstlimitierend verläuft und damit die Art des Schutzes (sterilisierend oder nur schützend vor symptomatischen Verläufen) nicht abschließend geklärt werden kann. Insgesamt kam es in der großen Phase-III-Studie von HEV-239 rechnerisch zu 4842 Infektionen der Kontrollgruppe, jedoch wurden – trotz engmaschiger Kontrollen – lediglich nur 1,1 % als Erkrankung erkannt. Diese asymptomatischen Infektionen können insbesondere bei immunsupprimierten Personen evtl. einen chronischen Verlauf nehmen und hier zu den relevanten Erkrankungen führen.^1^

Ein weiterer Ansatz der Protektion von Menschen in Deutschland vor einer HEV-Infektion ist die Verhinderung einer Übertragung des Erregers vom Hauptwirt (insbesondere Haus-, ggf. auch Wildschwein) auf den Menschen. Durch Immunisierungsstrategien bei den Tieren kann eine Reduktion des Anteiles HEV-positiver Nahrungsmittel erzielt werden. Dieser Ansatz steht im Mittelpunkt – bislang jedoch weniger – aktueller Forschungsansätze (diskutiert in Referenz [36]).

### Die Rolle zellulärer Immunität in der Protektion gegenüber HEV.

Insbesondere Personen während oder nach einer immunsuppressiven Therapie haben das Risiko der Entwicklung einer chronischen Verlaufsform der HEV-Infektion. Hierbei kann meist im Verlauf der Infektion Anti-HEV-IgG im Plasma detektiert werden. Zudem korreliert eine Anti-HEV-IgG-Positivität vor Infektionsgeschehen nicht unmittelbar mit dem Schutz vor der Entwicklung einer chronischen Infektion [37]. Wie auch bei anderen Erregern einer viralen Hepatitis stellt die zelluläre und insbesondere T‑zell-vermittelte Immunantwort einen entscheidenden Faktor in der Kontrolle der Infektion dar. Dies konnte sowohl für immunkompetente als auch für Personen mit Immunsuppression aufgezeigt werden [38, 39]. Zukünftige Impfstudien sollten daher insbesondere die zelluläre Immunität analysieren, um hier weitere Informationen über die Protektion gegenüber HEV-Infektionen ableiten zu können.

### Das ORF3 als Targetprotein – ein neuer Ansatz?

In den letzten Jahren konnte gezeigt werden, dass das Hepatitis-E-Virus während des Austrittes aus der infizierten Zelle die sogenannte ESCRT-Maschinerie nutzt („endosomal sorting complexes required for transport“). Dadurch wird das Virus mit einer Pseudoumhüllung ausgeschleust [40]. Dies hat zur Folge, dass im Blut von infizierten Personen die pseudoumhüllte Form des Virus nachgewiesen wird, während sich im Stuhl nach Verdauung der Hülle lediglich das nackte Virus befindet [41]. Bereits vor einem Jahrzehnt hat sich gezeigt, dass mit Serum von infizierten Personen in Zellkultur trotz Zugabe von neutralisierenden Antikörpern eine Infektion etabliert werden konnte, was am ehesten der Pseudoumhüllung zuzuschreiben ist [42]. Es bleibt bislang unklar, inwieweit diese Umhüllung das Virus vor neutralisierenden Antikörpern *in vivo* schützt.

Das ORF3-Protein, welches bezüglich der AS mit dem ORF2 überlappt, scheint struktureller Bestandteil der pseudoumhüllten Viren zu sein, da Anti-ORF3-Antikörper in der Lage sind, diese Viruspartikel zu binden [40]. Dies rückt das ORF3-Protein als mögliches zukünftiges Target für Immunisierungsstrategien ins Blickfeld. Erste Daten eines GT4-basierten ORF3-Impfstoffkandidaten, welches fusioniert mit Interleukin-1‑β in *E. coli* exprimiert wurde, zeigte jedoch in Makaken nur eine partielle Protektion vor einer Infektion [43]. Hierbei ist zu beachten, dass in den Infektionsmodellen Viren aus Stuhlsuspensionen genutzt wurden, welche entsprechend keine Pseudoumhüllung besitzen. Erschwert wird dieser Ansatzpunkt auch durch die hohe Variabilität der unterschiedlichen GT im Bereich der ORF3-Genomsequenz, welche entsprechend möglicherweise die Bindung der generierten Antikörper an andere ORF3-Proteine vermindert.

### Die Rolle unterschiedlicher Kapsidvarianten.

Im Rahmen einer HEV-Infektion lassen sich sowohl glykosylierte als auch nichtglykosylierte ORF2-Proteine nachweisen. Lediglich die nichtglykosylierten Varianten stellen die infektiösen Partikel dar und die glykosylierte Variante wirkt am ehesten als „Köder“ („decoy“) für die Antikörperantworten [44, 45]. Insbesondere immungeschwächte Personen weisen oft erhöhte Level der glykosylierten Variante auf, was ein Risikofaktor für eine chronische Verlaufsform ist [46]. Bislang liegen keine Daten bezüglich einer Spezifizierung der Antikörperantworten nach Immunisierung in Hinblick auf die Bindung von glykosylierten oder nichtglykosylierten Proteinen vor. Zukünftig könnte diese detaillierte Untersuchung der Antikörperantwort insbesondere für Impfungen gegen GT3 zum Schutz vor einer chronischen Infektion sinnvoll sein.

### Entwicklung neutralisierender Antikörper zur passiven Immunisierung.

Die bislang durchgeführten klinischen Studien des HEV-Vakzins zeigen auf, dass zumindest symptomatische Infektionen mit HEV verhindert werden können. Dies korreliert gut mit den Antikörperantworten auf das jeweilige Vakzin, sodass neutralisierenden Antikörpern in der Infektabwehr eine zentrale Rolle zugeschrieben werden kann. Dieses bildet die wissenschaftliche Grundlage aktueller Bestrebungen, Antikörper zu identifizieren, die pangenotypisch HEV neutralisieren [47]. Vergleichbar mit der passiven Impfung gegen Hepatitis A könnte dieses Prinzip als zukünftige passive Immunisierungsstrategie z. B. bei Personen nach Reisen in Endemiegebiete oder aber vulnerablen Personen (z. B. Immunsupprimierten) Anwendung finden.

## Fazit

Aufgrund des relevanten Beitrages der Hepatitis E zu Mortalität und Morbidität weltweit besteht ein hoher Bedarf an einem effektiven HEV-Vakzin. Die genotypische Heterogenität der HEV-Infektion in Bezug auf klinischen Verlauf, Übertragung und Risikogruppen stellt die Impfstoffentwicklung vor große Herausforderungen. Das ist der Grund, weshalb das Vakzin HEV-239, das an einer sehr großen Personengruppe evaluierte wurde, bislang lediglich eine Zulassung in China besitzt. Die aktuell in Entwicklung befindlichen Vakzinkandidaten haben das Ziel, eine protektive Antikörperantwort im Impfling zu induzieren. Bei der Analyse des Virus konnten kürzlich mehrere Mechanismen identifiziert werden, um eben dieser Immunantwort entgegenzuwirken. Zukünftige Impfstoffe sollten in ihrer Evaluation daher insbesondere diese Heterogenität und Abwehrmechanismen berücksichtigen. Zudem wäre die Entwicklung einer passiven Immunisierungsstrategie für vulnerable Personen mit eingeschränktem Immunsystem wünschenswert.

### Infobox 1 Vor- und Nachteile des HEV-239-Vakzins

**Pro**

- Impfung gegen HEV insgesamt erstrebenswert (WHO)
- Evaluation von HEV-239 in einer großen Phase-III-Studie mit hoher Patientenzahl
- Sehr gute Verträglichkeit
- Effektiver Schutz vor symptomatischen Infektionen
- Immunogen auch in älteren Personen (> 65 Jahren)
- Phase-IV-Studien mit Nachweis der Möglichkeit eines verkürzten, effektiven Impfschemas → evtl. Möglichkeit der Impfung von Reisenden

**Kontra**

- Kein Nachweis des Schutzes gegenüber anderen HEV-Genotypen als GT4
- Unklarheit bzgl. Immunogenität in Risikogruppen (insbesondere Schwangere, Personen mit chronischer Lebererkrankung, Immunsupprimierte, Reisende)
- Keine Daten für Personen < 16 Jahren
- Unklarheit bzgl. Ausmaß des Schutzes (sterilisierend vs. Schutz vor symptomatischer Infektion)
- Bislang keine Untersuchungen bei HEV-Ausbrüchen

## Footnotes

1 Vgl. hierzu Statement und Bewertung von Wenzel & Jilg (Referenzlabor für Hepatitis E, Universität Regensburg) auf der Webseite der Gesellschaft der Virologie (GfV) (https://www.g-f-v.org/node/679; Impfung gegen Hepatitis E: Ergebnisse der ersten Studie zur Langzeit-Wirksamkeit des neuen Impfstoffes, Februar 2016; Zugriff 10/2021).
